# Psychosocial correlates of unintentional weight loss in the second half of life in the German general population

**DOI:** 10.1371/journal.pone.0185749

**Published:** 2017-10-02

**Authors:** André Hajek, Jens-Oliver Bock, Hans-Helmut König

**Affiliations:** Department of Health Economics and Health Services Research, Hamburg Center for Health Economics, University Medical Center Hamburg-Eppendorf, Hamburg, Germany; Raddboudumc, NETHERLANDS

## Abstract

**Background:**

Unintentional weight loss (UWL) is common in older age and associated with adverse outcomes including mortality. The aim of the present study was to determine psychosocial correlates of UWL.

**Methods:**

Data were derived from a large, nationally representative study of community-dwelling individuals in the second half of life (40 years and over) in Germany in 2014 (n = 7,933). Data on UWL were assessed in face-to-face interviews as unintentional loss of more than 5kg (11 pounds) in weight in the past 12 months, and data on psychosocial factors were recorded in self-administered questionnaires.

**Results:**

Multiple logistic regressions revealed that UWL was positively associated with depressive symptoms and positive affect, whereas it was negatively associated with self-esteem. Individuals with UWL were more likely to feel lonely and perceive themselves as socially excluded.

**Conclusion:**

The findings of important psychosocial correlates of UWL may help to identify individuals at risk for UWL in older age. This is in particular important since interventions to treat this phenomenon in older age are available that reduce adverse consequences resulting from UWL.

## Introduction

Unintentional weight loss (UWL, also known as involuntary weight loss) is a common phenomenon in old age. Its prevalence is estimated to be between 15% and 20% in the older population above the age of 65 years [1]. UWL is associated with higher levels of morbidity [2] and with increased subsequent all-cause mortality [3]. Due to these severe adverse consequences, the causes of UWL have been widely investigated. Thus, previous studies have acknowledged that a broad variety of causes can be identified as potential UWL in old age [4]. Many studies pointed out organic causes as one main reason for UWL [5–11]. Among them, cancer and in particular gastrointestinal malignancy may cause UWL, followed by other gastrointestinal disorders including swallowing disorders and cardiac diseases. In addition, the role of psychological or psychiatric causes like depression and dementia has been acknowledged as causes of UWL. Yet a substantial number of cases of UWL remain unclear in clinical praxis [1, 2, 4, 12]. As an explanation of this unexplained UWL, psychosocial causes have been discussed [1, 2, 4, 12].

Yet previous studies investigating associations of UWL with various determinants, including some psychological or social variables, were restricted to small sample sizes in rather specific settings (as, for example an inpatient setting [9]) [1, 5–9, 11, 13] or they focused on rather nutrition-related correlates instead of psychosocial variables [10]. Consequently, evidence of psychosocial correlates of UWL, analyzed in the general older population, is sparse. However, this knowledge can be crucial to identify and characterize individuals at risk. This is particularly important since specific interventions intending to prevent the potential serious consequences from UWL are available [12]. Thereby, it depends on the cause of UWL whether, for example, nutritional interventions or pharmacological treatments may be appropriate.

Against the background of sparse knowledge about psychosocial correlates of UWL, the aim of this study was to identify psychosocial correlates of UWL in a large representative sample of community-dwelling older Germans at the age of 40 years and older. We examined comprehensively associations of psychological factors (depressive symptoms, positive and negative affect, life satisfaction, and self-esteem) and social factors (perceived social exclusion, loneliness, satisfaction with tight relationships), resorting to well-established constructs and measures. Thus, important associations of UWL may be revealed, providing new insights to the nature of this phenomenon.

Concerning the psychosocial factors, we hypothesized that UWL is associated with increased depressive symptoms. This might be explained by the fact that depressive symptoms are often accompanied by, for example, the loss of appetite [4]. Furthermore, we hypothesized that UWL is associated with decreased positive, and increased negative affect. A possible explanation might be that positive (e.g., active) and negative affects (e.g., nervous, upset, ashamed) are associated with food intake [14]. In addition, we hypothesized that UWL is associated with decreased life satisfaction. A possible explanation might be that individuals with high life satisfaction have a greater will to live [15]. This factor is in turn linked to the willingness to eat [16]. We also hypothesized that UWL is associated with low self-esteem. This is conceivable as low self-esteem is related to impaired self-care activities [17] which in turn are associated with UWL [18]. Moreover, we hypothesized that UWL is associated with increased social exclusion and loneliness. This might be explained by the fact that these factors are associated with malnutrition and insufficient caloric intake [19]. Lastly, we hypothesized that UWL is associated with low satisfaction with close relationships. This might be explained by the fact that food intake of, for example, physically impaired older individuals, might rely on friends or acquaintances.

## Methods

### Study population

The analysis is based on data form the German Ageing Survey (DEAS; Deutscher Alterssurvey), which is a longitudinal nationwide representative survey for the target population of community-dwelling adults aged 40 years and older. By means of national probability sampling techniques, individual addresses from registry offices in several German communities were randomly selected and corresponding citizens were offered participation via mail. Baseline recruitment was in 1996, and follow-ups took place in 2002, 2008, 2011, and in 2014. Thereby, each follow-up comprised a panel-sample of participants who had taken part before in the study and a newly drawn cross-sectional sample. The current study used data from the fifth wave only, because the outcome measure was only assessed in this wave. The instruments used in the fifth wave are available in German and English [20, 21].

For the follow-up in 2014, the ‘panel-sample’ consisted of 4,352 participants, corresponding to a response rate of 62.6%. Main reasons for not participating anymore in the study for the remaining 37.4% of all potential participants were a general withdrawal of consent to participate in DEAS (18.2%), no time (5.2%), and illness (5.1%). For the cross-sectional sample, the response rate was 28.4%, leading to a total of n = 6,090 participants. Among the 71.6% who refused participation, 28.7% did so for principal reasons, 15.1% had no time, 12.5% were not interested in the study, and 4.1% felt too ill to participate.

In 2014, the participants took part in face-to-face interviews about diverse topics on aging at their homes through computer assisted personal interviewing (CAPI). After the interview, the participants were asked to fill in a self-administered questionnaire that covered more personal topics like attitudes, preferences, and some psychological factors. While the variable ‘weight loss’ was assessed in the face-to-face interviews, data on important covariates for this study, like loneliness or social exclusion, were collected in the self-administered questionnaire. Therefore, the analyses for this study are based on the subsample with self-administered questionnaire with known status of UWL (n = 7,933). Prior to the interview, written informed consent was given. Explicit assurance to respondents was given that participation is voluntary. Prior to the study, participants were informed in written form about the aims and methods of this study. Moreover, they were informed in written form about the fact that the German Ageing Survey is in compliance with the Federal Data Protection Act.

In the first two waves (1996 and 2002) the participants took part in face-to-face interviews through paper assisted personal interviewing (PAPI). Since 2008, the interviews were conducted using CAPI. However, the response rate did not change. For example, the response rate for the cross-sectional sample was 34.9% in 2002 and 37.8% in 2008.

Please note that an ethical statement for this study was not necessary because criteria for the need of an ethical statement were not met (risk for the respondents, lack of information about the aims of the study, examination of patients). For example, invasive methods were not used.

Fieldwork was conducted by infas Institute for Applied Social Sciences. Infas only delivers survey data (without contact details) to the German Centre of Gerontology. At the German Centre of Gerontology, data are anonymized by the Research Data Centre (FDZ-DZA). Subsequently, the FDZ-DZA provides access and support to scholars interested in using DEAS for their research.

### Measures

#### Dependent variable: UWL

Data on weight loss were collected in face-to-face interviews at participants’ homes. They were confronted with the question: “Have you lost more than 5 kg [11 pounds] in body weight in the last year?” If a participant gave a positive answer, he or she was asked to report whether this has been intentionally or *un*intentionally.

#### Independent variables: Psychosocial factors

The individual items and construction of the psychological and social constructs are shown in detail in the S1 Table. Depressive symptoms were assessed using a 15-item short form of the translated version of the Center for Epidemiologic Studies Depression scale (CES-D). Cronbach’s alpha in our sample was 0.87. Life satisfaction was measured by the well-established Satisfaction with Life Scale [22]; Cronbach’s alpha equaled to 0.86 in our study. Positive affect and negative affect were assessed using the often used and validated Positive and Negative Affect Schedule (PANAS) [23]. Cronbach’s alpha was 0.87. Self-esteem was assessed by the Rosenberg scale [24]; Cronbach’s alpha was 0.84.

With respect to the social measures, perceived social exclusion was measures using a scale developed by Bude and Lantermann [25] whose Cronbach’s alpha was .88. A six-item scale assessed loneliness [26] with a Cronbach’s alpha of 0.83 in our sample. Satisfaction with the relationship with friends and acquaintances was quantified in a single item with five levels: “How would you rate your present relationship with your friends and acquaintances?” (from 1 = very good to 5 = very bad).

#### Independent variables: Socio-demographics, income and illness level

Beside age in years and gender, the marital status was included, reported as (i) ‘married and living together with spouse’ (reference category), (ii) ‘married and not living together with spouse’, (iii) ‘divorced’, (iv) ‘widowed’, and (v) ‘single’. The assessment of the family status depends on whether the individual has already taken part before or not. For the cross-sectional sample, married individuals and individuals living in civil union could report whether they lived together with spouse/registered partner or not. Informed by the German law (§ 1567 German Civil Code, BGB), individuals were assigned to “not living together with spouse” when the marriage/civil union has broken down irretrievably. Other married individuals and individuals living in civil union were classified as ‘married and living together with spouse’ including cases where the spouse/registered partner currently does not live in the same household for health or occupational reasons. For the panel sample, family status was quantified using changes in marital status (e.g., death of the spouse, divorcement).

The rare categories (civil union, living together, n = 19; civil union, living separated, n = 1; civil union, dissolved, n = 1; civil union, deceased, n = 0) were assigned to these categories (here: married and living together with spouse; married and not living together with spouse) for data security reasons.

The net monthly equivalent income was considered. The illness level was operationalized as number of diseases. The list of diseases that defined the sum score include (i) cardiac and circulatory disorders, (ii) bad circulation, (iii) joint, bone, spinal or back problems, (iv) respiratory problems, asthma, shortness of breath, (v) stomach and intestinal problems, (vi) cancer, (vii) diabetes, (viii) gall bladder, liver or kidney problems, (ix) bladder problems, (x) insomnia, (xi) eye problems, vision impairment, (xii) ear problems, hearing problems.

### Statistical analyses

Differences between the two groups of people with UWL and those without were analyzed by t-tests for metric variables and Chi^2^ tests for proportions. In addition, multiple logistic regressions were conducted. The statistical significance was determined with p<0.05. Stata 14.0 (StataCorp, College Station, Texas, USA) was used to perform analyses. The psychological explanatory variables were inserted separately into the model because of the degree of collinearity and were analyzed in terms of significance and effect sizes. What is more important, the key reason why the psychosocial factors were included separately in the regression models was to demonstrate that whenever *one* of these psychosocial factors is available, upcoming studies of UWL should include this factor as an independent variable. It was initially hypothesized that each of these psychosocial factors is significantly associated with UWL after adjustment for a full set of potential confounders such as socioeconomic variables or factors related to the lifestyle. As the percentage of missing value was low, regression models were complete case analyses, a technique also known as ‘listwise deletion’.

Based on previous studies and theoretical arguments, explanatory variables were selected [4, 27–29]. As explanatory variables, we included gender, age, marital status (Ref.: married living together with spouse; divorced; widowed; single), monthly net equivalence income in €1,000, and the number of chronic illnesses in all regression models. The other explanatory variables (depressive symptoms; life satisfaction; positive affect; negative affect; self-esteem; social exclusion; loneliness; satisfaction with the relationship with friends and acquaintances) were entered separately in the regression models, resulting in eight regression models. Further details (e.g., additional analysis) were described in the results section.

## Results

### Sample characteristics by experience of UWL

Sample characteristics stratified by experience of UWL are depicted in Table 1. 6.9% of the individuals experienced an UWL. In the total sample (n = 7,933), mean age was 64.5 years (SD: 11.2 years), ranging from 40 to 95 years.

**Table 1:** Sample characteristics by UWL (n = 7,933).

	Persons who did not experience an UWL (n = 7,388; 93.1%)		Persons who experienced an UWL (n = 545; 6.9%)		p-value	Missing values
	N/Mean	%/(SD)	N/Mean	%/(SD)		%
Gender: Female	3,741	50.6%	304	55.8%	0.02	0.0%
Age in years	64.45	(11.20)	65.66	(11.78)	0.02	0.0%
Marital status					<0.001	0.2%
married and living together with spouse’	5,223	70.8%	325	59.6%		
married and living separated from spouse	119	1.6%	9	1.7%		
divorced	720	9.8%	67	12.3%		
widowed	796	10.8%	95	17.4%		
single	514	7.0%	49	9.0%		
Monthly net equivalence income (€)	1959.30	(1387.56)	1689.85	(1228.28)	<0.001	5.6%
Number of physical illnesses	2.55	(1.86)	3.36	(2.04)	<0.001	1.5%
Loneliness	1.77	(0.54)	1.87	(0.57)	<0.001	1.9%
Life satisfaction	3.82	(0.72)	3.62	(0.84)	<0.001	1.0%
Positive affect	3.56	(0.53)	3.42	(0.57)	<0.001	1.2%
Negative affect	2.09	(0.52)	2.16	(0.57)	<0.01	1.1%
Self-esteem	3.40	(0.41)	3.29	(0.44)	<0.001	0.2%
Social exclusion	2.59	(0.58)	2.76	(0.68)	<0.001	1.4%
Satisfaction with relationship with friends and acquaintances	1.87	(0.59)	1.89	(0.66)	.65	1.7%
Depressive symptoms	6.41	(5.75)	9.98	(7.82)	< .001	1.8%

Loneliness (De Jong Gierveld & Van Tilburg, 2006); Life satisfaction (SWLS, Pavot & Diener, 1993); Positive and negative affect (PANAS, Watson et al., 1988); Self-esteem (Rosenberg, 1965); Depressive symptoms (CES-D, Hautzinger and Bailer, 1993); Social exclusion (Bude & Lantermann, 2006).

55.8% of the individuals who experienced an UWL were female compared to 50.6% of those who did not experience an UWL. 17.4% of the first group were widowed, compared to 10.8% of the latter group. Compared with individuals experiencing an UWL, their counterparts were younger, had a higher income, fewer physical diseases, less depressive symptoms, a higher satisfaction with life, a higher positive affect, less negative affect, higher self-esteem, felt less socially excluded, and had a lower loneliness score. In total, individuals with UWL had less favorable levels of almost all independent variables than those who did not experience an UWL.

It has been shown that, for example, the t-test is robust to deviations from normality if sample sizes are not very small [30]. However, we also analyzed bivariate differences with non-parametric tests, which can be considered as a conservative approach. Consequently, we also used the Mann-Whitney U (Wilcoxon rank sum) test instead of the t-test for independent samples. In terms of significance, results remained virtually the same (please see S2 Table for further details).

### Inferential analysis

Table 2 shows the results of multiple logistic regression models with UWL as dependent variables. All eight models are fully adjusted for the socio-demographic variables, income, and number of diseases. Model 1 to model 5 shows the results of the psychological variables, model 6 to model 8 those of the social factors. Thus, model 1 shows the relationship of depressive symptoms with UWL, which is positive and significant. Controlling for the same potential confounders, life satisfaction is negatively associated with UWL. Equally, positive affect is negatively associated with UWL, whereas negative affect was not associated with UWL. Self-esteem was significantly lower for individuals with UWL.

**Table 2:** Factors associated with UWL: Results of multiple logistic regressions.

Independent variables	(1) UWL	(2) UWL	(3) UWL	(4) UWL	(5) UWL	(6) UWL	(7) UWL	(8) UWL
Female (Ref. Male)	1.09 (0.90–1.32)	1.19+ (0.99–1.44)	1.20+ (0.99–1.45)	1.16 (0.96–1.40)	1.19+ (0.98–1.43)	1.18+ (0.97–1.42)	1.21+ (1.00–1.46)	1.17 (0.97–1.42)
Age in years	1.00 (0.99–1.01)	1.00 (0.99–1.01)	0.99 (0.99–1.00)	1.00 (0.99–1.01)	1.00 (0.99–1.01)	1.00 (0.99–1.01)	1.00 (0.99–1.01)	0.99 (0.99–1.00)
Marital status: Married, living separated from spouse (Ref.: married, living together with spouse)	1.19 (0.59–2.40)	1.19 (0.59–2.39)	1.29 (0.64–2.58)	1.28 (0.64–2.58)	1.27 (0.63–2.55)	1.10 (0.53–2.30)	1.24 (0.62–2.49)	1.33 (0.66–2.66)
Divorced	1.26 (0.94–1.70)	1.30+ (0.97–1.74)	1.40* (1.05–1.86)	1.41* (1.06–1.87)	1.39* (1.04–1.85)	1.36* (1.02–1.81)	1.38* (1.03–1.84)	1.46** (1.09–1.94)
Widowed	1.53** (1.16–2.02)	1.57** (1.20–2.07)	1.60*** (1.22–2.10)	1.60*** (1.22–2.11)	1.61*** (1.22–2.11)	1.62*** (1.24–2.13)	1.64*** (1.25–2.15)	1.57** (1.19–2.07)
Single	1.49* (1.06–2.09)	1.43* (1.02–2.00)	1.52* (1.09–2.11)	1.56** (1.12–2.17)	1.48* (1.06–2.06)	1.47* (1.06–2.06)	1.52* (1.09–2.12)	1.55* (1.11–2.16)
Monthly net equivalence income in €1,000	0.90* (0.82–1.00)	0.89* (0.81–0.99)	0.89* (0.80–0.98)	0.87** (0.78–0.96)	0.88* (0.80–0.97)	0.89* (0.80–0.98)	0.87** (0.78–0.96)	0.86** (0.78–0.95)
Number of chronic illnesses	1.13*** (1.07–1.19)	1.20*** (1.14–1.26)	1.20*** (1.14–1.26)	1.21*** (1.15–1.27)	1.19*** (1.13–1.25)	1.20*** (1.14–1.26)	1.20*** (1.15–1.26)	1.20*** (1.15–1.26)
Depressive symptoms	1.06*** (1.05–1.08)							
Life satisfaction		0.81** (0.71–0.92)						
Positive affect			0.71*** (0.59–0.85)					
Negative affect				1.08 (0.91–1.29)				
Self-esteem					0.68*** (0.55–0.85)			
Social exclusion						1.27** (1.10–1.47)		
Loneliness							1.22* (1.03–1.44)	
Satisfaction with the relationship with friends and acquaintances								1.02 (0.88–1.19)
Constant	0.03*** (0.01–0.05)	0.11*** (0.05–0.22)	0.20*** (0.08–0.48)	0.05*** (0.02–0.11)	0.20*** (0.08–0.52)	0.03*** (0.01–0.06)	0.04*** (0.02–0.08)	0.06*** (0.03–0.12)
Observations	7,242	7,287	7,278	7,280	7,341	7,269	7,237	7,229
Pseudo R^2^	0.05	0.04	0.04	0.03	0.04	0.04	0.04	0.03

Observations with missing values were dropped (listwise deletion). Loneliness (De Jong Gierveld & Van Tilburg, 2006); Life satisfaction (SWLS, Pavot & Diener, 1993); Positive and negative affect (PANAS, Watson et al., 1988); Self-esteem (Rosenberg, 1965); Depressive symptoms (CES-D, Hautzinger and Bailer, 1993); Social exclusion (Bude & Lantermann, 2006). Odds ratios were reported; 95% confidence intervals in parentheses;

*** p<0.001,

** p<0.01,

* p<0.05,

^+^ p<0.10;

With respect to the social factors, perceived social exclusion and loneliness were increased in participants with UWL. In contrast to that, the satisfaction with the relationship to friends was unrelated to UWL.

We also stratified our regression analysis by age (individuals younger than 65 years; individuals at least 65 years; please see S3 and S4 Tables for further details). While most of the results were comparable (in terms of significance and effect size) to regression analysis conducted for the total sample, some differences are worth noting. UWL was significantly associated with life satisfaction and self-esteem among individuals younger than 65 years, whereas it was not significantly associated with these explanatory variables among older individuals (with life satisfaction, p = .08; with self-esteem, p = .29). Contrarily, UWL was significantly associated with increased loneliness among older individuals, whereas loneliness was not associated with UWL in individuals younger than 65 years (p = .59).

It might be the case that not all somatic diseases included in the variable “number of chronic illnesses” are associated with UWL. Consequently, additional regression analysis was performed with physical illnesses known to be associated with UWL, i.e. (1) cardiac and circulatory disorders, (2) respiratory problems, asthma, shortness of breath, (3) stomach and intestinal problems, (4) cancer, and (5) gall bladder, liver or kidney problems [9, 29]. In terms of effect sizes and significance, the associations between UWL and psychosocial factors remained virtually the same. Furthermore, increased UWL was associated with the presence of cardiac and circulatory disorders, stomach and intestinal problems, cancer, and gall bladder, liver or kidney problems, whereas it was not associated with respiratory problems, asthma, and shortness of breath. Further details are provided in S5 Table.

## Discussion

### Main findings

The aim of this study was to determine and analyze psychosocial correlates of UWL in the general German population above the age of 40 years. Multiple regression analyses showed that UWL was positively associated with the psychological variables of depressive symptoms, and negatively with those of life satisfaction, positive affect and self-esteem. Among the social determinants, perceived social exclusion and loneliness were associated with increased likelihood of UWL. Negative affect and satisfaction with the relationship to friends remained insignificant in the multiple logistic regressions.

### Previous research

Our cross-sectional study has found that depressive symptoms are associated with UWL. Previous studies have shown that depressive symptoms are associated with UWL [6, 8, 9, 11, 13, 31]. This was, for example, found by Schilp et al., showing that undernutrition (defined as either a BMI < 20 kg/m^2^ or self-reported involuntary weight loss ≥ 5% in the last 6 months) was associated with more depressive symptoms [32]. However, the study by Schilp et al. did not distinguish between UWL and BMI < 20 kg/m^2^. A possible explanation for the relationship between UWL and depressive symptoms is that the latter one is often associated with loss of appetite or reduced motivation to prepare food [4].

In the present study, positive affect was negatively associated with UWL, whereas negative affect was only associated with UWL in pairwise comparisons. Consequently, after controlling for several covariates such as family status or the number of chronic diseases the association between negative affect and UWL disappeared. Thus, the relation between negative affect and UWL might be explained, at least in part, by family status and chronic illnesses. Actually, when we did not adjust for family status and chronic illnesses, the association between UWL and NA was significant (OR: 1.31, 95%-CI: 1.10–1.55, p = .002; Pseudo-R^2^ = .01).

We found that satisfaction with life was negatively associated with UWL. Generally, individuals scoring high in life satisfaction tend to be more hopeful about the future [33]. Thus, they might have a greater ‘will to live’ [15] which in turn might be associated with food intake as well as the willingness to ‘force’ themselves to eat as discussed by Wikby and Fägerskiöld [16]. Consequently, it appears plausible that life satisfaction is negatively related with UWL.

UWL was negatively associated with self-esteem in our study. This might be explained by the fact that low self-esteem is associated with impaired self-care activities [17] such as taking medications, performing self-examinations or food records. It is assumed that these self-care activities are associated with UWL [18].

Using a study at 11 sites in Europe (Czech Republic, Denmark, Finland, Iceland, Norway, Sweden, Netherlands, France, Italy, Germany, and UK; stratified random sample with n = 4,010), Sørbye et al. showed that there was a positive association between UWL and feeling lonely (OR = 1.4; CI (95%): 1.1–1.8), reduced social activity (OR: 2.6; CI (95%): 2.1–3.2), and not being out of house in the last week (OR: 1.4; CI (95%): 1.1–1.8) among individuals aged 65 and over who received home-care services [10]. This is in accordance with our study where loneliness and social exclusion were found to be positively associated with UWL. In some cases, food intake of physically impaired individuals might mainly rely on irregular visits by *family members or caregivers*. When they additionally suffer from social exclusion or loneliness, insufficient food intake as well as malnutrition might be the consequence [19]. Nevertheless, in our study, UWL was *not* significantly related to the satisfaction with relationship with friends and acquaintances. A possible explanation might be that food intake of these individuals did not rely on *friends or acquaintances*.

Regarding the remaining control variables, our findings are mostly in accordance with previous studies. For example, several studies found that chronic diseases such as cancer are positively associated with UWL [5–7, 9, 31]. Furthermore, it has been found that this outcome measure is associated with income or loss of the spouse [27, 34].

### Strengths and limitations

A strength of the present study was that the data were derived from a nationally representative study of individuals (≥ 40 years) living in private households located in Germany. In addition, widely established scales were used to assess psychological factors examined in our study. Furthermore, various potential confounders such as sociodemographic factors and number of diseases were included in the study. Limitations of the present study include the cross-sectional design, which does not allow for the inference of a cause-and-effect association. Furthermore, the analysis is restricted to the UWL greater than 5 kg. Future studies are necessary to examine the impact of other cut-offs or using relative percentages. Yet, similar cut-offs are widely used [1, 35, 36] and in accordance with the frailty guidelines proposed for shrinking by Fried et al [37]. Furthermore, even though a sample selection bias cannot be ruled out, it is supposed to be rather small [38, 39] because participants only slightly differ from individuals not taking part [40]. While, for example, nationality and age were associated with the probability of taking part, region and age of the interviewer were not associated with this probability. This sample selection bias might result in the fact that it might be difficult to generalize our findings to, for example, individuals in very old age. In addition, satisfaction with the relationship with friends or acquaintances was assessed using a single item-scale, which raises the question of the reliability. However, it has been demonstrated that these single-items scales measuring domain satisfaction have satisfactory psychometric properties [41]. Moreover, we cannot dismiss the possibility that the use of listwise deletion is a limitation of the current study. However, the proportion of missing data is quite low in this study, indicating that this bias might be quite small [42].

## Conclusion

Using a population-based study of individuals in the second half of life, factors associated with UWL were identified. The analyses showed several important psychosocial concepts associated with UWL. This knowledge may help to identify individuals at risk for UWL in older age and enhance the knowledge of the nature of this frequent phenomenon in older age. For example, it might be a good idea to gather information on psychosocial factors routinely when general practitioners are consulted by older patients. Future research with longitudinal study designs is necessary to gain insights into the causal relationship between psychosocial factors and UWL.

## Supporting information

S1 Table Psychological factors (items and explanations). Items with asterisk have been recoded.
(DOCX)

S2 Table Sample characteristics by UWL (n = 7,933). Loneliness (De Jong Gierveld & Van Tilburg, 2006); Life satisfaction (SWLS, Pavot & Diener, 1993); Positive and negative affect (PANAS, Watson et al., 1988); Self-esteem (Rosenberg, 1965); Depressive symptoms (CES-D, Hautzinger and Bailer, 1993); Social exclusion (Bude & Lantermann, 2006). P-values are based on the Mann-Whitney U (Wilcoxon rank sum) test (instead of the t-test for independent samples).
(DOCX)

S3 Table Factors associated with UWL: Results of multiple logistic regressions among individuals younger than 65 years. Odds ratios were reported; 95% confidence intervals in parentheses; *** p<0.001, ** p<0.01, * p<0.05, + p<0.10; Observations with missing values were dropped (listwise deletion). Loneliness (De Jong Gierveld & Van Tilburg, 2006); Life satisfaction (SWLS, Pavot & Diener, 1993); Positive and negative affect (PANAS, Watson et al., 1988); Self-esteem (Rosenberg, 1965); Depressive symptoms (CES-D, Hautzinger and Bailer, 1993); Social exclusion (Bude & Lantermann, 2006).
(DOC)

S4 Table Factors associated with UWL: Results of multiple logistic regressions among individuals at least 65 years. Odds ratios were reported; 95% confidence intervals in parentheses; *** p<0.001, ** p<0.01, * p<0.05, + p<0.10; Observations with missing values were dropped (listwise deletion). Loneliness (De Jong Gierveld & Van Tilburg, 2006); Life satisfaction (SWLS, Pavot & Diener, 1993); Positive and negative affect (PANAS, Watson et al., 1988); Self-esteem (Rosenberg, 1965); Depressive symptoms (CES-D, Hautzinger and Bailer, 1993); Social exclusion (Bude & Lantermann, 2006).
(DOC)

S5 Table Factors associated with UWL: Results of multiple logistic regressions. Odds ratios were reported; 95% confidence intervals in parentheses; *** p<0.001, ** p<0.01, * p<0.05, + p<0.10; Observations with missing values were dropped (listwise deletion). Loneliness (De Jong Gierveld & Van Tilburg, 2006); Life satisfaction (SWLS, Pavot & Diener, 1993); Positive and negative affect (PANAS, Watson et al., 1988); Self-esteem (Rosenberg, 1965); Depressive symptoms (CES-D, Hautzinger and Bailer, 1993); Social exclusion (Bude & Lantermann, 2006).
(DOC)
