# Supplementary material for: Psychosocial correlates of unintentional weight loss in the second half of life in the German general population
Source: PLoS One. 2017 Oct 2;12(10):e0185749. doi: 10.1371/journal.pone.0185749 (PMC5624619; doi:10.1371/journal.pone.0185749)
Supplement: S1 Table — Items with asterisk have been recoded. (DOCX) [file pone.0185749.s001.docx]

S1 Table: Psychological factors (items and explanations)

| Psychological factors | Items and explanations |
| --- | --- |
| Loneliness (De Jong Gierveld & Van Tilburg, 2006) | Emotional loneliness   1. I experience a general sense of emptiness. 2. I miss having people around. 3. I often feel rejected.   Social loneliness   1. There are plenty of people I can rely on when I have problems. (*) 2. There are many people I can trust completely. (*) 3. There are enough people I feel close to. (*)   Scale represents the mean of at least 3 required valid items, 3 items have been recoded.  1 = strongly agree  2 = agree  3 = disagree  4 = strongly disagree  High values represent high loneliness. |
| Life satisfaction (SWLS, Pavot & Diener, 1993) | 1. In most ways my life is close to my ideal. 2. The conditions of my life are excellent. 3. I am satisfied with my life. 4. So far I have gotten the important things I want in life. 5. If I could live my life over, I would change almost nothing.   Scale represents the mean of at least 3 required valid items, all items have been recoded.  1 = strongly agree  2 = agree  3 = neither agree nor disagree  4 = disagree  5 = strongly disagree  High values represent high life satisfaction. |
| Negative affect (PANAS, Watson et al., 1988) | In the following you will find a number of words that describe different feelings and emotions. Please indicate to what extent you have felt this way during the past few months.   1. Distressed 2. Upset 3. Guilty 4. Scared 5. Hostile 6. Irritable 7. Ashamed 8. Nervous 9. Jittery 10. Afraid   Scale represents the mean of at least 3 required valid items.  1 = very slightly or not at all  2 = a little  3 = moderately  4 = quite a bit  5 = extremely  High values on the NA scale represent high frequency of negative emotions. |
| Positive affect (PANAS, Watson et al., 1988) | In the following you will find a number of words that describe different feelings and emotions. Please indicate to what extent you have felt this way during the past few months.   1. Enthusiastic 2. Excited 3. Strong 4. Interested 5. Proud 6. Alert 7. Inspired 8. Determined 9. Attentive 10. Active   Scale represents the mean of at least 3 required valid items.  1 = very slightly or not at all  2 = a little  3 = moderately  4 = quite a bit  5 = extremely  High values on the PA scale represent high frequency of positive emotions. |
| Self-esteem (Rosenberg, 1965) | 1. On the whole, I am satisfied with myself. (*) 2. I am able to do things as well as most other people. (*) 3. All in all, I am inclined to feel that I am a failure. 4. I feel that I have a number of good qualities. (*) 5. I certainly feel useless at times. 6. I feel that I‘m a person of worth, at least on an equal plane with others. (*) 7. I feel I do not have much to be proud of. 8. I take a positive attitude toward myself. (*) 9. I wish I could have more respect for myself. 10. At times I think I am no good at all.   Scale represents the mean of at least 3 required valid items, 5 items have been recoded.  1 = strongly agree  2 = agree  3 = disagree  4 = strongly disagree  High values represent high self-esteem. |
| Social exclusion (Bude and Lantermann, 2006) | 1. I am worried to be left behind. (*) 2. I feel like I do not really belong to society. (*) 3. I feel that I am left out. (*) 4. I feel excluded from society. (*)   Scale reflects the mean of at least two required (recoded) valid items.  1 = strongly agree  2 = agree  3 = disagree  4 = strongly disagree  Higher values represent high perceived social exclusion. |

Legend: Items with asterisk have been recoded.
