# Supplementary material for: Psychosocial correlates of unintentional weight loss in the second half of life in the German general population
Source: PLoS One. 2017 Oct 2;12(10):e0185749. doi: 10.1371/journal.pone.0185749 (PMC5624619; doi:10.1371/journal.pone.0185749)
Supplement: S2 Table — Loneliness (De Jong Gierveld & Van Tilburg, 2006); Life satisfaction (SWLS, Pavot & Diener, 1993); Positive and negative affect (PANAS, Watson et al., 1988); Self-esteem (Rosenberg, 1965); Depressive symptoms (CES-D, Hautzinger and Bailer, 1993); Social exclusion (Bude & Lantermann, 2006). P-values are based on the Mann-Whitney U (Wilcoxon rank sum) test (instead of the t-test for independent samples). (DOCX) [file pone.0185749.s002.docx]

**S2 Table.** Sample characteristics by UWL (n=7,933)

|  | Persons who did not experience an UWL  (n=7,388; 93.1%) | | Persons who experienced an UWL (n=545; 6.9%) | | p-value | Missing values |
| --- | --- | --- | --- | --- | --- | --- |
|  | N/Mean | %/(SD) | N/Mean | %/(SD) |  | % |
| Gender: Female | 3,741 | 50.6% | 304 | 55.8% | <0.001 | 0.0% |
| Age in years | 64.45 | (11.20) | 65.66 | (11.78) | <0.01 | 0.0% |
| Marital status |  |  |  |  | <0.001 | 0.2% |
| married and living together with spouse’ | 5,223 | 70.8% | 325 | 59.6% |  |  |
| married and living separated from spouse | 119 | 1.6% | 9 | 1.7% |  |  |
| divorced | 720 | 9.8% | 67 | 12.3% |  |  |
| widowed | 796 | 10.8% | 95 | 17.4% |  |  |
| single | 514 | 7.0% | 49 | 9.0% |  |  |
| Monthly net equivalence income (€) | 1959.30 | (1387.56) | 1689.85 | (1228.28) | <0.001 | 5.6% |
| Number of physical illnesses | 2.55 | (1.86) | 3.36 | (2.04) | <0.001 | 1.5% |
| Loneliness | 1.77 | (0.54) | 1.87 | (0.57) | <0.001 | 1.9% |
| Life satisfaction | 3.82 | (0.72) | 3.62 | (0.84) | <0.001 | 1.0% |
| Positive affect | 3.56 | (0.53) | 3.42 | (0.57) | <0.001 | 1.2% |
| Negative affect | 2.09 | (0.52) | 2.16 | (0.57) | <0.05 | 1.1% |
| Self-esteem | 3.40 | (0.41) | 3.29 | (0.44) | <0.001 | 0.2% |
| Social exclusion | 2.59 | (0.58) | 2.76 | (0.68) | <0.001 | 1.4% |
| Satisfaction with relationship with friends and acquaintances | 1.87 | (0.59) | 1.89 | (0.66) | .28 | 1.7% |
| Depressive symptoms | 6.41 | (5.75) | 9.98 | (7.82) | <.001 | 1.8% |

Loneliness (De Jong Gierveld & Van Tilburg, 2006); Life satisfaction (SWLS, Pavot & Diener, 1993); Positive and negative affect (PANAS, Watson et al., 1988); Self-esteem (Rosenberg, 1965); Depressive symptoms (CES-D, Hautzinger and Bailer, 1993); Social exclusion (Bude & Lantermann, 2006). P-values are based on the Mann-Whitney U (Wilcoxon rank sum) test (instead of the t-test for independent samples).
