# Supplementary material for: Psychosocial correlates of unintentional weight loss in the second half of life in the German general population
Source: PLoS One. 2017 Oct 2;12(10):e0185749. doi: 10.1371/journal.pone.0185749 (PMC5624619; doi:10.1371/journal.pone.0185749)
Supplement: S3 Table — Odds ratios were reported; 95% confidence intervals in parentheses; *** p<0.001, ** p<0.01, * p<0.05, + p<0.10; Observations with missing values were dropped (listwise deletion). Loneliness (De Jong Gierveld & Van Tilburg, 2006); Life satisfaction (SWLS, Pavot & Diener, 1993); Positive and negative affect (PANAS, Watson et al., 1988); Self-esteem (Rosenberg, 1965); Depressive symptoms (CES-D, Hautzinger and Bailer, 1993); Social exclusion (Bude & Lantermann, 2006). (DOC) [file pone.0185749.s003.doc]

S3 Table. Factors associated with UWL: Results of multiple logistic regressions among individuals younger than 65 years
	(1)	(2)	(3)	(4)	(5)	(6)	(7)	(8)	
Independent variables	UWL	UWL	UWL	UWL	UWL	UWL	UWL	UWL	
									
Female (Ref. Male)	1.46**	1.54**	1.55**	1.52**	1.57***	1.60***	1.65***	1.56**	
	(1.10 - 1.92)	(1.18 - 2.02)	(1.19 - 2.03)	(1.16 - 1.99)	(1.20 - 2.05)	(1.22 - 2.09)	(1.26 - 2.16)	(1.19 - 2.05)	
Age in years	1.02+	1.02+	1.02	1.02+	1.02+	1.02*	1.03*	1.02+	
	(1.00 - 1.05)	(1.00 - 1.04)	(0.99 - 1.04)	(1.00 - 1.04)	(1.00 - 1.04)	(1.00 - 1.05)	(1.00 - 1.05)	(1.00 - 1.04)	
Marital status: Married, living separated from spouse (Ref.: married, living together with spouse)	2.56*	2.66*	2.84*	2.82*	2.68*	2.18	2.61*	3.06*	
	(1.03 - 6.36)	(1.08 - 6.53)	(1.16 - 6.95)	(1.15 - 6.89)	(1.10 - 6.54)	(0.83 - 5.71)	(1.07 - 6.39)	(1.25 - 7.51)	
Divorced	1.30	1.20	1.29	1.29	1.25	1.23	1.17	1.31	
	(0.82 - 2.07)	(0.76 - 1.91)	(0.82 - 2.04)	(0.82 - 2.03)	(0.79 - 1.98)	(0.78 - 1.95)	(0.74 - 1.86)	(0.83 - 2.07)	
Widowed	1.38+	1.43*	1.46*	1.45*	1.41*	1.41*	1.41*	1.37+	
	(1.00 - 1.90)	(1.04 - 1.97)	(1.06 - 2.00)	(1.05 - 1.99)	(1.03 - 1.94)	(1.03 - 1.93)	(1.03 - 1.94)	(0.99 - 1.90)	
Single	0.90	0.93	0.97	1.00	0.95	0.95	0.92	1.02	
	(0.43 - 1.91)	(0.46 - 1.89)	(0.48 - 1.96)	(0.49 - 2.02)	(0.47 - 1.92)	(0.47 - 1.92)	(0.45 - 1.86)	(0.50 - 2.06)	
Monthly net equivalence income in €1,000	0.91	0.91	0.90	0.89	0.89	0.90	0.89	0.89	
	(0.79 - 1.06)	(0.78 - 1.05)	(0.78 - 1.05)	(0.77 - 1.03)	(0.77 - 1.03)	(0.78 - 1.05)	(0.76 - 1.03)	(0.77 - 1.04)	
Number of chronic illnesses	1.07*	1.15***	1.15***	1.16***	1.14***	1.14***	1.14***	1.14***	
	(1.00 - 1.15)	(1.08 - 1.23)	(1.08 - 1.22)	(1.08 - 1.24)	(1.07 - 1.22)	(1.07 - 1.22)	(1.07 - 1.22)	(1.06 - 1.21)	
Depressive symptoms	1.07***								
	(1.05 - 1.09)								
Life satisfaction		0.85+							
		(0.70 - 1.02)							
Positive affect			0.77*						
			(0.59 - 0.98)						
Negative affect				1.03					
				(0.80 - 1.34)					
Self-esteem					0.84				
					(0.61 - 1.16)				
Social exclusion						1.27*			
						(1.04 - 1.56)			
Loneliness							1.33*		
							(1.05 - 1.68)		
Satisfaction with the relationship with friends and acquaintances								1.05	
								(0.85 - 1.31)	
Constant	0.01***	0.02***	0.03***	0.01***	0.02***	0.00***	0.00***	0.01***	
	(0.00 - 0.03)	(0.00 - 0.09)	(0.00 - 0.21)	(0.00 - 0.05)	(0.00 - 0.12)	(0.00 - 0.02)	(0.00 - 0.02)	(0.00 - 0.05)	
									
Observations	3,639	3,661	3,654	3,655	3,697	3,647	3,635	3,621	
Pseudo R²	0.0563	0.0353	0.0360	0.0335	0.0338	0.0369	0.0386	0.0298	
Odds ratios were reported; 95% confidence intervals in parentheses; *** p<0.001, ** p<0.01, * p<0.05, + p<0.10; Observations with missing values were dropped (listwise deletion). Loneliness (De Jong Gierveld & Van Tilburg, 2006); Life satisfaction (SWLS, Pavot & Diener, 1993); Positive and negative affect (PANAS, Watson et al., 1988);  Self-esteem (Rosenberg, 1965); Depressive symptoms (CES-D, Hautzinger and Bailer, 1993); Social exclusion (Bude & Lantermann, 2006).
