# Supplementary material for: Psychosocial correlates of unintentional weight loss in the second half of life in the German general population
Source: PLoS One. 2017 Oct 2;12(10):e0185749. doi: 10.1371/journal.pone.0185749 (PMC5624619; doi:10.1371/journal.pone.0185749)
Supplement: S5 Table — Odds ratios were reported; 95% confidence intervals in parentheses; *** p<0.001, ** p<0.01, * p<0.05, + p<0.10; Observations with missing values were dropped (listwise deletion). Loneliness (De Jong Gierveld & Van Tilburg, 2006); Life satisfaction (SWLS, Pavot & Diener, 1993); Positive and negative affect (PANAS, Watson et al., 1988); Self-esteem (Rosenberg, 1965); Depressive symptoms (CES-D, Hautzinger and Bailer, 1993); Social exclusion (Bude & Lantermann, 2006). (DOC) [file pone.0185749.s005.doc]

S5 Table. Factors associated with UWL: Results of multiple logistic regressions. 
	(1)	(2)	(3)	(4)	(5)	(6)	(7)	(8)	
Independent variables	UWL	UWL	UWL	UWL	UWL	UWL	UWL	UWL	
									
Female (Ref. Male)	1.09	1.18	1.19+	1.14	1.18	1.17	1.20+	1.16	
	(0.89 - 1.34)	(0.97 - 1.44)	(0.97 - 1.45)	(0.93 - 1.39)	(0.96 - 1.44)	(0.96 - 1.43)	(0.98 - 1.47)	(0.95 - 1.42)	
Age in years	1.00	1.00	1.00	1.00	1.00	1.00	1.00	1.00	
	(0.99 - 1.01)	(0.99 - 1.01)	(0.99 - 1.01)	(0.99 - 1.01)	(0.99 - 1.01)	(0.99 - 1.01)	(0.99 - 1.01)	(0.99 - 1.01)	
Marital status: Married, living separated from spouse (Ref.: married, living together with spouse)	1.27	1.25	1.37	1.35	1.34	1.15	1.30	1.39	
	(0.62 - 2.57)	(0.62 - 2.53)	(0.68 - 2.75)	(0.67 - 2.73)	(0.67 - 2.71)	(0.55 - 2.41)	(0.64 - 2.62)	(0.69 - 2.81)	
Divorced	1.32+	1.33+	1.44*	1.45*	1.43*	1.38*	1.42*	1.50**	
	(0.97 - 1.79)	(0.98 - 1.80)	(1.07 - 1.94)	(1.07 - 1.95)	(1.06 - 1.92)	(1.02 - 1.86)	(1.05 - 1.91)	(1.12 - 2.02)	
Widowed	1.55**	1.59**	1.61**	1.63**	1.64***	1.64***	1.68***	1.60**	
	(1.15 - 2.09)	(1.18 - 2.13)	(1.20 - 2.17)	(1.21 - 2.19)	(1.22 - 2.20)	(1.23 - 2.21)	(1.25 - 2.25)	(1.18 - 2.16)	
Single	1.52*	1.46*	1.56*	1.60**	1.51*	1.51*	1.55*	1.59*	
	(1.06 - 2.17)	(1.03 - 2.08)	(1.10 - 2.21)	(1.13 - 2.26)	(1.07 - 2.15)	(1.06 - 2.15)	(1.09 - 2.20)	(1.12 - 2.25)	
Monthly net equivalence income in €1,000	0.90*	0.89*	0.89*	0.86**	0.88*	0.89*	0.86**	0.86**	
	(0.82 - 1.00)	(0.80 - 0.99)	(0.80 - 0.98)	(0.78 - 0.96)	(0.80 - 0.98)	(0.80 - 0.98)	(0.78 - 0.96)	(0.77 - 0.95)	
Cardiac and circulatory disorders (Ref.: No)	0.80*	0.76**	0.76**	0.75**	0.77*	0.75**	0.76*	0.74**	
	(0.64 - 0.98)	(0.62 - 0.94)	(0.62 - 0.93)	(0.61 - 0.92)	(0.63 - 0.95)	(0.61 - 0.92)	(0.62 - 0.94)	(0.60 - 0.92)	
Respiratory problems, asthma, shortness of breath (Ref.: No)	1.04	0.93	0.94	0.92	0.95	0.95	0.95	0.90	
	(0.80 - 1.34)	(0.73 - 1.20)	(0.73 - 1.20)	(0.72 - 1.18)	(0.74 - 1.22)	(0.74 - 1.22)	(0.74 - 1.22)	(0.70 - 1.16)	
Stomach and intestinal problems (Ref.: No)	0.62***	0.56***	0.56***	0.56***	0.56***	0.57***	0.55***	0.58***	
	(0.49 - 0.79)	(0.44 - 0.71)	(0.44 - 0.71)	(0.44 - 0.70)	(0.45 - 0.71)	(0.45 - 0.72)	(0.43 - 0.69)	(0.45 - 0.73)	
Cancer (Ref.: No)	0.61**	0.57***	0.56***	0.56***	0.57***	0.57***	0.56***	0.55***	
	(0.45 - 0.82)	(0.43 - 0.76)	(0.42 - 0.75)	(0.42 - 0.75)	(0.42 - 0.76)	(0.42 - 0.76)	(0.42 - 0.75)	(0.41 - 0.74)	
Gall bladder, liver or kidney problems (Ref.: No)	0.57***	0.54***	0.53***	0.53***	0.53***	0.52***	0.51***	0.53***	
	(0.43 - 0.76)	(0.40 - 0.71)	(0.40 - 0.70)	(0.40 - 0.70)	(0.40 - 0.70)	(0.39 - 0.69)	(0.38 - 0.67)	(0.40 - 0.70)	
Depressive symptoms	1.06***								
	(1.05 - 1.08)								
Life satisfaction		0.80**							
		(0.70 - 0.92)							
Positive affect			0.70***						
			(0.58 - 0.84)						
Negative affect				1.16					
				(0.96 - 1.40)					
Self-esteem					0.64***				
					(0.51 - 0.81)				
Social exclusion						1.35***			
						(1.16 - 1.58)			
Loneliness							1.25*		
							(1.05 - 1.49)		
Satisfaction with the relationship with friends and acquaintances								1.01	
								(0.86 - 1.18)	
Constant	0.82	6.95**	13.84***	2.79	14.93***	1.65	2.58	4.60*	
	(0.23 - 2.92)	(2.09 - 23.11)	(3.75 - 51.11)	(0.71 - 10.95)	(4.05 - 55.02)	(0.44 - 6.16)	(0.72 - 9.22)	(1.32 - 15.96)	
									
Observations	6,756	6,804	6,797	6,797	6,850	6,793	6,760	6,741	
Pseudo R²	0.0643	0.0447	0.0459	0.0423	0.0462	0.0467	0.0457	0.0391	
Odds ratios were reported; 95% confidence intervals in parentheses; *** p<0.001, ** p<0.01, * p<0.05, + p<0.10; Observations with missing values were dropped (listwise deletion). Loneliness (De Jong Gierveld & Van Tilburg, 2006); Life satisfaction (SWLS, Pavot & Diener, 1993); Positive and negative affect (PANAS, Watson et al., 1988);  Self-esteem (Rosenberg, 1965); Depressive symptoms (CES-D, Hautzinger and Bailer, 1993); Social exclusion (Bude & Lantermann, 2006).
